# Scalar Implicatures: The Psychological Reality of Scales

**DOI:** 10.3389/fpsyg.2016.01500

**Published:** 2016-10-25

**Authors:** Alex de Carvalho, Anne C. Reboul, Jean-Baptiste Van der Henst, Anne Cheylus, Tatjana Nazir

**Affiliations:** ^1^CNRS, Institute for Cognitive Sciences-Marc Jeannerod (UMR5304), University Claude Bernard Lyon 1, BronFrance; ^2^Laboratoire de Sciences Cognitives et Psycholinguistique (ENS, EHESS, CNRS), Departement d’Etudes Cognitives, École Normale Supérieure, PSL Research UniversityParis, France

**Keywords:** lexical scales, masked priming, lexical decision task, scalar implicature, implication, experimental pragmatics, psycholinguistics

## Abstract

Scalar implicatures, the phenomena where a sentence like “The pianist played *some* Mozart sonatas” is interpreted, as “The pianist did *not* play *all* Mozart sonatas” have been given two different analyses. Neo-Griceans (NG) claim that this interpretation is based on lexical scales (e.g., <*some, all*>), where the stronger term (e.g., *all*) implies the weaker term (e.g., *some*), but the weaker term (e.g., *some*) implicates the negation of the stronger term (i.e., *some* = *not all*). Post-Griceans (PG) deny that this is the case and offer a context-based inferential account for scalar implicatures. While scalar implicatures have been extensively investigated, with results apparently in favor of PG accounts, the psychological reality of lexical scales has not been put to the test. This is what we have done in the present experiment, with a lexical decision task using lexical scales in a masked priming paradigm. While PG accounts do not attribute any role for lexical scales in the computation of scalar implicatures, NG accounts suggest that lexical scales are the core mechanism behind the computation of scalar implicatures, and predict that weaker terms in a scale should prime stronger terms more than the reverse because stronger words are necessary to the interpretation of weaker words, while stronger words can be interpreted independently of weaker words. Our results provided evidence in favor of the psychological existence of scales, leading to the first clear experimental support for the NG account.

## Introduction

The notion of *implicature* was introduced by Grice (1975) to account for information that was *communicated* without being, strictly speaking, *said* by the speaker, in other words, for information that was *implicitly* rather than *explicitly* communicated (Grice, 1989). For instance, if the speaker asked where Anne lives, an answer such as “Somewhere in Burgundy, I believe,” *conversationally implicates* that he does not know exactly where she lives.

Grice distinguished among conversational implicatures those that (as in the previous example) strongly depend on the **context** (the Particularized Conversational Implicatures: PCIs) from those that depend on the **words** used (the Generalized Conversational Implicatures: GCIs). The paramount examples of GCIs are so-called *scalar implicatures.* For instance, if the speaker says, “The pianist played *some* Beethoven sonatas,” she implicates, through the use of *some*, that the pianist did *not* play *all* of them. Note that both GCIs and PCIs are computed through the same mechanism: they are the result of an inference that was made by comparing what the speaker says with what she might have said but did not say. In other words, these inferences are based on alternatives to what was said. A sentence such as “Anne lives somewhere in Burgundy, I believe,” leads us to derive a PCI, i.e., the speaker does not know where exactly Anne lives, because we quickly infer that if he knew it, he would have said where she lives precisely. Through the same inferential mechanism a GCI such as “The pianist played *some* Beethoven sonatas,” implicates that he did not play all of them, because if he had played all of them (on the assumption that the speaker knew it), the speaker would have used *all* rather than *some*. However, despite the fact that they share the same inferential mechanism, PCIs and GCIs differ in the ways in which “the alternatives” are determined: through the **context** for PCIs and though the **lexicon** for GCIs.

### Neo-Gricean and Post-Gricean Accounts

The notion of implicature was quickly incorporated from philosophy of language into pragmatics, but led to two highly different approaches: The Neo-Gricean (NG) and the Post-Gricean (PG) approaches. The NG approaches (see e.g., Horn, 1972, 2004; Levinson, 2000; Chierchia, 2004) claimed, on the basis of scalar implicatures, that GCIs are derived locally and automatically (by default) when the trigger belongs to a linguistic *scale*. Such lexical scales are ordered sets of terms, such as <*and, or*>, where the stronger member, *and*, implies the weaker member, *or*, and the weaker member, *or*, implicates the negation of the stronger member: *p or q* implicates *not (p and q)* (i.e., one or the other but not both). The implicature interpretation can be canceled in favor of a semantic interpretation (*p or q and possibly both*), but this will only come after the pragmatic interpretation has been accessed and at a cost.

The NG approach is a more or less straightforward extension of Gricean theory, in the sense that it considers scalar implicatures to be conversational implicatures, and that it is a development of Grice’s intuition that some conversational implicatures are entirely dependent on the context while others are not (and scalar implicatures are a major example of the latter kind). Scalar implicatures—and this is the new development brought about by Horn (1972)—depends on the existence of lexical scales. Horn proposed that, in the case of scalar implicatures, the alternatives we compare to what the speaker said are determined by the lexical scale to which the term that triggers the inference belongs. In other words, when a weaker term of a scale (e.g., “some”) is used in a sentence, a comparison is made with the stronger term(s) in the scale (e.g., “all”) as alternatives to what the speaker said (e.g., “If she used *some*, it’s because it’s not *all*”). In a more recent development of this position, Levinson (2000) went one-step further and proposed that the pragmatic interpretation of the scalar term is *lexicalized as its default interpretation*. In other terms, the pragmatic interpretation of scalar items is encoded as a (defeasible) part of its meaning (i.e., “some” also means “not all”), while the semantic interpretation (i.e., “some = at least one”) would only be accessible if the pragmatic interpretation is explicitly negated (e.g., “The pianist played some Mozart sonatas and even all of them”).

By contrast, PG accounts, such as Relevance Theory (see e.g., Sperber and Wilson, 1986/1995; Carston, 2002), consider scalar implicatures to be explicatures rather than any kind of conversational implicatures. They result from a process of pragmatic enrichment of the linguistic interpretation of the utterance (the so-called logical form), yielding a relevant truth-conditional propositional form. This enrichment process is on a par with what happens for most utterances (e.g., loose talk, metaphors, etc). For instance, a sentence such as “This steak is raw,” uttered in restaurant, is usually interpreted as *This steak is undercooked*. This final interpretation is obtained through a contextually driven process of *ad hoc* concept construction (loosening or strengthening) applying locally to the concept raw^1^. In such cases, the *ad hoc* concept construction is not, in any sense, a lexically based process: it is a contextually driven non-linguistic, conceptual process. The claim that scalar implicatures are interpreted through an identical process of *ad hoc* concept construction excludes both any Gricean-style mechanism based on alternatives and any role for the lexical scales as proposed by NG approaches. Additionally, *ad hoc* concept construction is believed to be a cognitively costly process, which implies that scalar implicatures will come at a price and will be accessed only when the context makes them relevant (see Noveck and Sperber, 2007 for a discussion). Thus, the PG approach differs from NG approaches in that it gives a central place to context and sees scalar inferences as the result of a contextual process, not allowing any role to lexical scales.

Previous experimental work on scalar inferences has concentrated on the opposite predictions drawn from the two accounts regarding *processing cost*. According to the NG account, the pragmatic interpretation is less costly than the semantic interpretation. On the PG accoun, the semantic interpretation is less costly than the pragmatic interpretation. Cashing the notion of *cost* in terms of cognitive difficulty, this suggests that the most costly interpretation should come later in cognitive development and that it should take more time to be processed. Thus, NG predicts that the *semantic* interpretation should come later and take more time, while PG predicts that it is the *pragmatic* interpretation that should come later and take more time. Studies that contrasted NG and PG accounts in terms of processing cost have provided robust evidence in favor of the PG account, because there is a clear progression of pragmatic interpretations from the younger age to adults (Noveck, 2001; see also Papafragou and Musolino, 2003; Papafragou and Tantalou, 2004; Guasti et al., 2005; Pouscoulous et al., 2007) and reaction time (RT) measures in adults show that pragmatic interpretations of scalar terms take longer to access than semantic interpretations (Bott and Noveck, 2004; Bott et al., 2012 but see for different results Feeney et al., 2004). Moreover, the proportion of pragmatic answers observed with adults was strongly context-dependent (see also Hartshorne et al., 2015 and Dupuy et al., 2016, for more data on the strong context-sensitivity of pragmatic interpretations for scalar implicatures). This context-dependency contradicts Levinson’s default account, which implies that all underinformative sentences with scalar terms should be given pragmatic interpretations and that semantic interpretations should only be given when the implicature is explicitly negated.

Thus, all the experimental results up to now strongly favor PG accounts and starkly contradict the predictions of NG accounts. There is nevertheless a crucial and interesting element in NG that has not been empirically investigated: the psychological reality of lexical scales.

### Current State of the Debate

While the simple lexical default account proposed by Levinson (2000) has been definitely contradicted by the experimental evidence, a new and more sophisticated NG account has recently been proposed by Chierchia (2013) and has not yet been tested. Chierchia proposes a far-ranging theory, encompassing not only scalars, but free-choice implicatures, polarity items, as well as upward and downward entailing linguistic environments. Regarding scalar implicatures, Cherchia argues that they result from a covert exhaustification operator (roughly equivalent in meaning to *only*) that operates on a set of alternatives determined by the scale the scalar term belongs to. However, this set of alternatives is only available to the exhaustification process if the context makes it mandatory to derive the implicature. For instance, if, in answer to the question “Did the pianist play *all* Mozart sonatas?”, the speaker hearer answers “He played *some* Mozart sonatas,” the alternative set including *most* and *all* will be available, while if the question had been “Did the pianist play Mozart sonatas?”, it would not be. Chierchia (2013, p. 104) notes that relevance to conversational goals is the central contextual factor in the derivation of scalar inferences.

On this new version of NG, quite a few of the differences with PG disappear: Chierchia does not commit himself about the cost of the implicature. He acknowledges a major role of the context, including what he calls “conversational relevance,” which determines whether or not the scalar inference will be drawn. However, in Chierchia’s theory, the alternatives are entirely due to Horn scales (e.g., <*all, many, some*>, which are lexically determined). It is the psychological reality of such scales that we are interested in testing in the present study.

There is no question that words inside a scale usually form a ‘family’ in the sense that they have related meanings (e.g., <*all, many, some*>) are all quantifiers. On this, both NG and PG would agree, but there is more to a scale than words with related meanings. In the NG account, the stronger words in a scale (i.e, “all”) are necessary for the interpretation of the weaker words (i.e., “some”) whenever an implicature is derived (they yield the alternative set: e.g., ‘some and maybe all’), while the stronger words can be interpreted without recourse to the weaker words in all circumstances (e.g., ‘all’ is always all, not less). These two characteristics of scales —that words inside a scale are related, and that there is an interpretive *asymmetry* due to the fact that stronger words are necessary to the interpretation of the weaker words, but the reverse is not true— open a road for behavioral investigations, using a masked priming paradigm (Forster and Davis, 1984).

As scales are supposed to be recovered automatically from the lexicon in NG (the context makes them available or not to the exhaustification mechanism), the simple and automatic nature of masked priming in a lexical decision task seems particularly appropriate to test the question of the psychological reality of Horn scales. Given that one form of priming is semantic in nature (i.e., words belonging to the same semantic fields prime one another more strongly than they prime words from other semantic fields (Perea and Rosa, 2002), we expect that words belonging to the same scale should prime one another. Crucially, as scales are ordered sets of words and given the NG notion that the stronger words are used in the interpretation of the weaker words, while the stronger words can be interpreted regardless of the weaker words, there should be an *asymmetry* in priming: weaker words in a scale should prime stronger words in the same scale more than stronger words would prime weaker words. For instance, in the scale <*all, many, some*>, *some* should prime *many* and *all* more than *many* would prime *some* and more than *all* would prime *many* and *some*.

By contrast, given that PG does not give lexical scales any role in the construction of the *ad hoc* concepts that it sees as the core of scalars, at most it would predict that, as any set of semantically related words, words inside a scale would prime one another more strongly than they would prime other words. However, it would not predict any asymmetry in the strength of priming between weaker and stronger words.

## Experiment: Lexical Decision Task with Masked Priming

In order to test the asymmetry prediction, a lexical decision task with masked priming was conducted. The masked priming paradigm (e.g., Forster and Davis, 1984) consists in presenting a subliminal prime to facilitate the processing of a target word. Note that priming is the phenomenon by which the presentation of a first item (the prime) will influence the processing of a second item (the target). In masked priming, the prime is presented subliminally, that is, too quickly for the participant to be aware that it was presented. These priming paradigms with a simple lexical decision task (where participants have to decide whether the target is a word or a non-word, after they have been presented with another word subliminally) give us a good opportunity to test the psychological reality of scales. Hence, this is a simple experimental paradigm that does not depend on any kind of reasoning and that is largely automatic given that the prime is not consciously perceived (Dehaene et al., 1998).

In particular, participants were presented with a subliminal prime word followed by the target and asked to judge whether the target was a word or a non-word. The measure was the RT between the presentation of the target and the participant’s answer. The task included two experimental conditions: in one condition, the prime was a weaker term than the target on the informativity scale (Implicature condition: e.g., *SOME — all*); in the other condition the prime was a stronger member than the target (Implication condition: e.g., *ALL — some*). Additionally, two control conditions were designed: one in which the prime and the target were identical (Identical condition: e.g., *SOME — some*); and one in which the prime was a sequence of consonants of the same length (in terms of number of letters) as the target (Consonant condition: e.g., *ZSQW — some*).

The identical condition should yield the shortest average RT because a term maximally primes itself. The consonant condition should have the longest RT response, because there cannot be any priming effect at all in this condition. Thus, these two control conditions should allow us to verify that the experiment worked well and to have a control on whether or not the RT of the participants is the result of the simple processing and reading of the target stimuli. Regarding the experimental conditions, the NG account (which supposes the psychological reality of scales) predicts that the target should be evaluated faster in the implicature condition (e.g., SOME – all) than in the implication condition (e.g., ALL – some).

## Materials and Methods

### Participants

Participants were 48 French native speakers, graduate students from the Ecole Normale Supérieure in Lyon, aged 20–30, right-handed, with normal or corrected-to-normal vision (20 males, mean age 22.4; 28 females, mean age 21.4). They participated on a voluntary basis, with no financial compensation. Five additional participants were tested but their data were not included in our analysis because they were ambidextrous (3), or because they made more than 30 errors (10%) during the test (2).

### Design and Stimuli

The experimental material was built on the basis of 129 items: 43 scalar terms, 43 pseudo-words and 43 sequences of consonants. We tested 18 scales: 11 included two words (e.g., <*and, or*>) and 7 three words (e.g., <*some, most, all*>) (c.f., Supplementary Data Sheet 1 for a complete list of scales). Middle words from the three-word scales were used for both the implication and implicature conditions. The scales we tested were chosen among those mentioned in the NG literature (e.g., Levinson, 2000; Horn, 2004). Given that our purpose was to test the general hypothesis that priming effect would be stronger in the implicature than in the implication condition, we took scales from various syntactic categories, connectives, quantifiers, adverbs, verbs, and adjectives, and scales composed of two or three words, without assuming any particular difference between them. This choice was motivated by the fact that we did not have any specific hypothesis on whether these different categories would trigger stronger or weaker effects of priming or on whether the number of lexical items in the scale (two or three) would modulate the priming effect. French words belonging to scales were used and were controlled for length and frequency of word, letters, bigram and trigram with the LEXIQUE database (New et al., 2004). The pseudo-words were created with an application from the Lexique Toolbox, which is a generator of pseudo-words from the same database. The pseudo-words were controlled for length and bigram frequency. Crucially, note that the frequency of the target words used had a similar range between the two experimental conditions (implication condition: mean = 1.81, median = 1.88, *SD* = 1.10, range: from -0.47 to 4.13; implicature condition: mean = 2.18; median = 1.98, *SD* = 1.24, range: from 0.49 to 4.32).

Each target word in the scales was either primed with itself, its matching consonant or the other word(s) in the scale, resulting in 150 prime-target stimuli (11^∗^2^∗^3 + 7^∗^3^∗^4) and 150 matching pseudo-words conditions for a total of 300 trials. Thus, each word was seen by each subject, as a prime and as a target in the identical condition, as a prime or as target in the implicature and implication conditions, as a prime for the pseudo-word condition and as a target in the consonant condition (where the sequence of consonants was used as a prime). For a better understanding of the way that the words were assigned to the different conditions presented to participants, Supplementary Data Sheet 1 provides a table of stimuli showing for each target word, the words presented as a prime in each condition.

The entire list of stimuli were presented in a fully within-subjects design, such that all subjects saw exactly the same stimuli in each condition in a different randomized order, for each subject.

### Procedure

**FIGURE 1:**
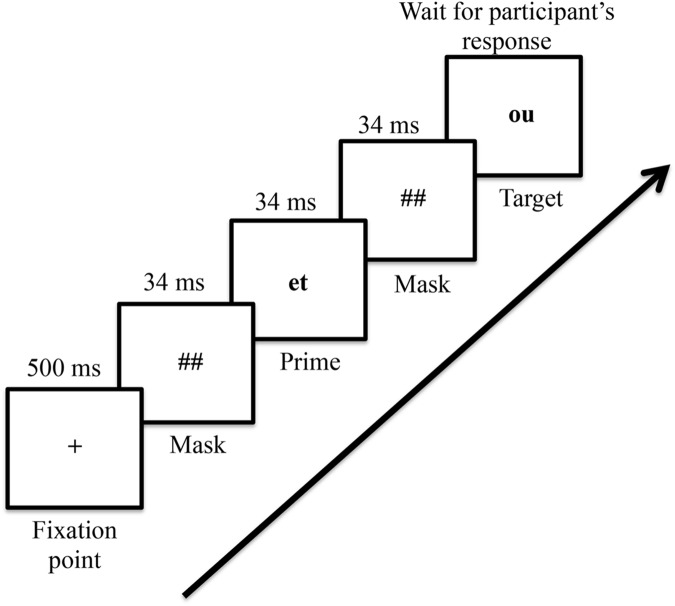
**Time course for a single trial.** Each trial started with a fixation point in the middle of the screen presented during 500 ms. Then a mask, a prime and a mask appeared for 34 ms each, immediately followed by the target word that appeared in the screen until the participant answered if it was a word or a pseudo-word. Note that for each trial, the mask contained exactly the same numbers of characters of the word used as prime.

The experiment was implemented with Neurobehavioural Systems, Inc. Presentation^®^ 14.9 program. The experiment took place on an individual basis in a quiet experimental room. Each trial started with a fixation point presented in the center of the screen for 500 ms. Then a forward mask (######) was presented for 34 ms and was immediately followed by a uppercase prime presented for 34 ms. The prime was replaced by another mask (######) for 34 ms before the target appeared on the screen. Participants were instructed to press one of two pre-defined buttons on the keyboard (the ‘right’ and the ‘left’ key buttons) to indicate whether the lower case letter string was a French word or not. For half of the participants the ‘right’ key corresponded to the ‘yes’ response and for the other half to the ‘no’ response. The target remained on the screen until participant’s response (see **Figure 1**). The lexical decision had to be performed as rapidly and as accurately as possible. The dependent variables were the RTs and error rates. When the participant responded, the target disappeared from the screen. The inter-trial interval was 1500 ms. Participants were not informed of the presence of the prime and in a debriefing after the experiment, none of them have reported detecting the prime words during the experiment.

### Data Analyses

Statistical data analysis and graphics were produced with R software version 3.2.2 (R Core Team, 2015) with packages multcomp (Hothorn et al., 2008) and lme4 (Bates et al., 2015). The response time analysis included only correct answers (per subject average 97.83%, median = 98.00%; range: from 93.00 to 99.67%). RTs below 300 ms and above 2000 ms were automatically excluded from the analysis because we assume that responses longer than 2000 ms reflect distraction rather than lexical decision and responses below 300 ms reflect anticipatory responses prior to proper stimulus processing. For the remaining trials, RTs outside of the interval defined by the intra-subject average ±2.5 standard deviation were discarded to minimize the impact of outliers on mean RT. Using these procedures, 5.58% of the initial data were discarded from the final analysis. RTs were then averaged for each participant in each of the different conditions prior to the calculation of the grand average over all participants.

## Results

**FIGURE 2:**
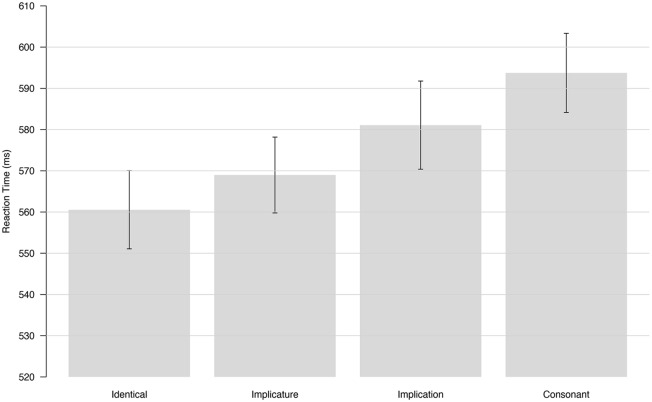
**Grand Average of Reaction Time (RT; ms) obtained for each condition.** Error bars represent the standard error of the mean across 48 subjects.

**Figure 2** shows the averaged RTs for each condition and the standard error across all participants. Average RTs for the target words presented in the identical condition were faster than average RTs for the same targets in the consonant condition. Average RTs for the two experimental conditions were in-between these two control conditions with higher averaged RTs in the implication condition than in the implicature condition.

**FIGURE 3:**
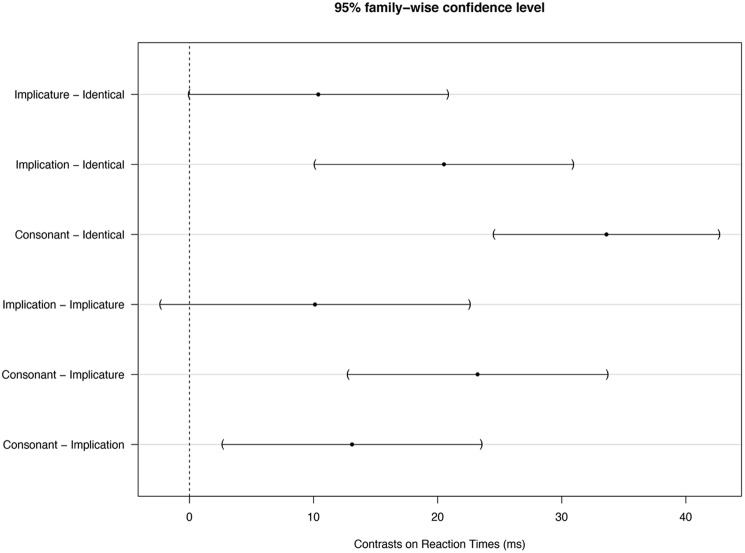
**Confidence intervals obtained with a 95% family wise estimate for Tukey contrasts on condition with our linear mixed effect model**.

Note, however, that the target words in the implicature condition (e.g., *SOME —* ***all***) and implication conditions (e.g., *ALL —* ***some***) are not the same. Potential differences in RTs between these two conditions could therefore be related to differences in the default reading time of the target words themselves. We therefore used a linear mixed effect model to analyze our data, with condition as a fixed effect and target word and participants as random effects. Confidence intervals for Tukey contrasts estimated with this model and a 95% family wise confidence level are shown in **Figure 3.** Tests that these contrasts are null based on the model are reported in **Table 1.**

**Table 1:** Simultaneous tests for general linear hypotheses estimated with a linear mixed effect model with condition as a fixed effect and subject and target word as random effects.

	Estimate	Standard error	*z*-value	Pr(>|t|) single step	Pr(>|t|) Uncorrected
Implicature – Identical = = 0	10.386	4.071	2.551	0.05154	0.01074
Implication – Identical = = 0	20.506	4.062	5.048	<0.001	<0.001
Consonant – Identical = = 0	33.606	3.542	9.489	<0.001	<0.001
Implication – Implicature = = 0	10.119	4.861	2.082	0.15689	0.03738
Consonant – Implicature = = 0	23.220	4.081	5.689	<0.001	<0.001
Consonant – Implication = = 0	13.100	4.070	3.219	0.00703	0.00258


*The *p*-values reported in Pr(>|t|) columns are adjusted either with a single-step method or no correction.*

**Figure 3** and **Table 1** indicate an estimated 10.12 ms reduction of response time in the implicature condition compared with the implication condition. Confidence intervals based on a 95% family wise confidence level slightly overlaps with zero and the single-step *p*-value adjustment indicates that this effect is not significant (*p* = 0.16). Note, however, that we are only interested in the single contrast between Implication and Implicature conditions. Correction for multiple testing is therefore not required. The uncorrected *p*-value is significant (*p* = 0.04). The data can thus be interpreted in favor of the existence of scales.

Following reviewers’ suggestions, we also looked at the data scale by scale. As our data does not allow us to conduct statistical analysis (since for each subject we had only one data point of RT for each scale in each condition), this analysis is presented as “Supplementary Presentation 1” to which the interested reader is directed.

## Discussion

As reviewed in our introduction, all the experimental literature has favored PG over NG accounts of how scalar implicatures are derived. However, one issue that has not been experimentally investigated so far is the psychological reality of lexical scales, a central issue for NG. Additionally, given recent developments in NG accounts (see Chierchia, 2013), the existence of scales has become the main point of departure between NG and PG, or at least one that is open to behavioral measures.

Using a masked priming paradigm, we tested the differential predictions of the two accounts. Predictions, based on the NG account, were that RTs in the implicature condition would be faster than in the implication condition because weaker words of a scale should prime stronger words of the same scale more than stronger words prime weaker words. By contrast, following the PG account, one would expect words inside a scale to prime one another (due to their syntactic and semantic proximity), but no such asymmetry of priming would be predicted, as scales are not supposed to play any role in the derivation of pragmatic interpretations for scalar implicatures.

The experiment described in this paper shows that an asymmetric relation holds between the members of lexical scales implicated in scalar implicature computations: weaker terms of a scale (e.g., “seldom”) primed stronger terms (e.g., “never”) more than the reverse. In a word decision task with masked priming, where participants were asked to judge whether the target presented in a screen was or was not a word in French, they were faster to judge that the stronger term of a scale was a word when it was subliminally preceded by the weaker term of the scale (e.g., “*SELDOM – never*”), than to judge that the weaker term of the scale was a word when it was subliminally preceded by the stronger term (e.g., “*NEVER – seldom*”). This asymmetry suggests, for the first time in the literature, that lexical scales are a psychological reality.

These results do also allow us to distinguish between the different predictions of the two main accounts of the role of lexical scales in the generation of pragmatic interpretation for scalar implicatures. They clearly favor the involvement of scales in the derivation of the pragmatic interpretation for scalar implicatures, in keeping with NG predictions and in contradiction with PG predictions.

Our results do not address, however, two further questions. The first one concerns the diversity of the scales we tested. As it can be seen in Supplementary Data Sheet 1, we have tested a somewhat heterogeneous set of lexical scales and it is possible that some scales would induce pragmatic interpretations at a much higher rate than others would. As suggested by the reviewers of this paper, the effect generated by lexical scales with logical connectives (e.g., <*and, or*>), quantifiers (e.g., <*all, many, some*>) and modals (<*allowed, obligatory*>) could be stronger than other scales such as <*bright, intelligent*>. Supporting this hypothesis, Van Tiel et al. (2016) have argued, based on experimental investigations, that some scales (notably <*all, many, some*> and <*and, or*>) induce a much higher rate of pragmatic interpretations for scalar implicatures than do others (e.g., <*small, tiny*>). Although we have checked our results by scale and observed that our significant priming effect from Implication minus Implicature conditions is observed for the majority of the scales we tested, independently of the type of lexical scale or the number of items it contains (see “Supplementary Presentation 1” for an exploratory analysis by scale), we did not conduct statistical analyses using “scale” as a factor, because for each subject we had only one data point per scale in each condition. So this analysis would be meaningless. Consequently, our data does not allow us to propose an interpretation of the effects derived by each scale individually. However, it might be considered that since the overall pattern of results can be observed for the majority of the scales we tested; despite of their heterogeneity, the asymmetry in RT between the implication and the implicature conditions seems to be robust enough. Further investigations using the same methodology exploited in this paper (masked priming) could, however, be done to address the question of the differences in the magnitude of the effect across scales. For instance, it would be important to investigate more precisely whether the variability between scales that has been evidenced in recent work using other experimental methods (see Van Tiel et al., 2016) and seems to be present in our data (see “Supplementary Presentation 1”), could be replicated in other studies with specific predictions about how and why some lexical scales can behave differently in they way they induce pragmatic interpretations. Nevertheless, our results are entirely compatible with the idea that scales may differ in how strongly they mandate pragmatic interpretations, or in the degree of automaticity with which they are accessed in the interpretation of scalar implicatures.

The second question that our results do not address concerns the possibility for participants to consider alternatives beyond the lexical items that appear in Horn scales, as it was recently suggested in a computational model of pragmatic inferences developed by Peloquin and Frank (2016). Their model tried to account for the fact that people consider the use of “*some*” inappropriate when the speaker could have used “*one*” or “*two*” and for the fact that when asked to produce alternative words to replace a word in a sentence (e.g., “*some*” in “*Some students came”*), people come up with lexical items beyond the relevant Horn scale (e.g., not only “*many*” or “*all*,” but also “*few*” and “*none*”). This led to the proposition that the alternative set for “*some*” should include “*none*,” “*few*,” “*most*,” “*all*”. We think that this does not pose a major problem for Horn scales: the first phenomenon does not lead to a pragmatic interpretation but merely to an infelicity judgment, which does not necessarily entail a pragmatic interpretation to “*not some*”; the second phenomenon does not seem to have anything to do with the derivation of a pragmatic interpretation. So we take it that the present results should be interpreted, quite simply, as a way of adjudicating between the two main approaches to scalar implicatures.

This is thus one of the first empirical results clearly consistent with the new version of NG account as recently proposed by Chierchia (2013). It should not, however, be taken to verify it in its entirety. The process through which scalar implicatures are derived in that account is complex. Additionally, the whole account is wide ranging and cannot be reduced to the interpretation of scalars. However, we provide an important first step in the empirical investigation of that account and bring a new type of data examining to what extent different words on lexical scales prime one another, which allowed us to distinguish accounts of scalar implicature generation.

Note, however, that the present results are obtained from words presented in isolation, while pragmatic interpretations are obtained for scalar terms occurring in sentences, usually in context. Our results do not have much to say about the process itself (notably they shed no light on whether as claimed by Chierchia (2013), it is an exhaustification process using a silent operator on sets of alternatives). However, they strongly suggest, given the asymmetry in RTs between the implication and implicature conditions, that scales must play a role in the interpretation process of scalar implicatures. Otherwise, the asymmetry would not have been observed. It is, indeed, hard if not impossible to explain this asymmetry based on the PG account. Finally, it should be noted that the involvement of scales in the interpretation process of scalar implicatures, which is the conclusion mandated by our experimental results, is compatible not only with Chierchia’s (2013) syntactic NG approach, but also with pragmatic Gricean approaches (Geurts, 2009; Geurts and Pouscoulous, 2009; Geurts and Van Tiel, 2013).

In summary, this study reported the first experimental evidence leading a distinction between the two main accounts for the derivation of pragmatic interpretations for scalar implicatures: *NG* versus *PG*. While PGs refuse any role for lexical scales in the derivation of scalar inferences and offer a context-based inferential account for scalar implicatures, NG accounts claim that lexical scales are the core mechanism behind the computation of scalar implicatures, and predict an asymmetry in priming between the implicature and the implication conditions. Supporting this hypothesis, the results that we obtained in a lexical decision task using lexical scales in a masked priming paradigm showed that weaker terms in a scale primed stronger terms more than the reverse. This asymmetry provides then the first experimental evidence in favor of the psychological existence of scales and therefore supports the claim of NG accounts for the role of lexical scales in the computation of scalar implicatures.

## Author Contributions

AdC and AR have designed the study and written the paper. AC programmed the experiment. AdC ran the experiments and analyzed the data under the supervision of TN, AC and J-BV.

## Conflict of Interest Statement

The authors declare that the research was conducted in the absence of any commercial or financial relationships that could be construed as a potential conflict of interest.

## Footnotes

1 As is usual, small capitals are used here for concepts, not for words.
