# Supplementary material for: Scalar Implicatures: The Psychological Reality of Scales
Source: Front Psychol. 2016 Oct 25;7:1500. doi: 10.3389/fpsyg.2016.01500 (PMC5078746; doi:10.3389/fpsyg.2016.01500)
Supplement: Supplementary file 1 [file Data_Sheet_1.pdf]

Appendix 1: List of scales tested

Scales

<un, le>

a, the

<ou, et>

or, and

<peu, aucun>

few, none

<rarement, jamais>

seldom, never

<près, à>

near, at

<certains,beaucoup, tous>

some, many, all

<parfois, fréquemment, toujours>

sometimes, often, always

<possible, probable, certain>

possible, likely, certain

<impossible, improbable, incertain>

impossible, unlikely, uncertain

<pouvoir, devoir>

can, must

<croire, savoir>

to believe, to know

<autoriser, obliger>

to allow, to force

<autorisé, obligatoire>

allowed, obligatory

<intelligent, brillant>

intelligent, bright

<tiède, chaud, bouillant>

warm, hot, boiling

<frais, froid, glacial>

cool, cold, frosty

<acceptable, bon, excellent>

acceptable, good, excellent

<approcher, atteindre>

to approach, to reach

Appendix 2: List of stimuli. Each row represents a target word used in the experiment and each column shows the prime used for this word in each condition

TARGET	Prime in the IDENTICAL condition	Prime in the IMPLICATION condition	Prime in the IMPLICATURE condition	Prime in the CONSONANT condition
un	UN	LE		FV
le	LE		UN	QZ
ou	OU	ET		DK
et	ET		OU	RK
peu	PEU	AUCUN		KTR
aucun	AUCUN		PEU	TDPRZ
rarement	RAREMENT	JAMAIS		SQRGTBPK
jamais	JAMAIS		RAREMENT	KPTMLN
près	PRÈS	À		XWZR
à	À		PRÈS	Q
pouvoir	POUVOIR	DEVOIR		NQPTXRK
devoir	DEVOIR		POUVOIR	CXWRTP
croire	CROIRE	SAVOIR		KDSRFBCX
savoir	SAVOIR		CROIRE	ZKXTPB

autoriser	AUTORISER	OBLIGER		KSBTGBTRM
obliger	OBLIGER		AUTORISER	ZRTPMKG
autorisé	AUTORISÉ	OBLIGATOIRE		WXCVBNKG
obligatoire	OBLIGATOIRE		AUTORISÉ	GFMNBRSXKTP
intelligent	INTELLIGENT	BRILLANT		QDSFGKMRZTX
brillant	BRILLANT		INTELLIGENT	TRZPFDKQ
approcher	APPROCHER	ATTEINDRE		TBCZNPQMK
atteindre	ATTEINDRE		APPROCHER	QNXSCVKPR
certains	CERTAINS	BEAUCOUP / TOUS		PKRFZQNB
beaucoup	BEAUCOUP	TOUS	CERTAINS	RXTZKMNQ
tous	TOUS		BEAUCOUP / CERTAINS	MZXR
parfois	PARFOIS	FRÉQUEMMENT / TOUJOURS		ZKRXMQP
fréquemment	FRÉQUEMMENT	TOUJOURS	PARFOIS	KFQDSZRTPVN
toujours	TOUJOURS		FRÉQUEMMENT / PARFOIS	VGTRDXSZ
possible	POSSIBLE	PROBABLE / CERTAIN		SDFKNBVT
probable	PROBABLE	CERTAIN	POSSIBLE	ZCPTKFSNMQD
certain	CERTAIN		PROBABLE / POSSIBLE	ZSQWXCV
impossible	IMPOSSIBLE		IMPROBABLE / INCERTAIN	MLKPTRZQGT
improbable	IMPROBABLE	IMPOSSIBLE	INCERTAIN	RDKPBGVFCD
incertain	INCERTAIN	IMPROBABLE / IMPOSSIBLE		LKJFTRSND
tiède	TIÈDE	CHAUD / BOUILLANT		RTKZB
chaud	CHAUD	BOUILLANT	TIÈDE	NBVCX
bouillant	BOUILLANT		CHAUD / TIÈDE	RFVTGBPKN
frais	FRAIS	FROID / GLACIAL		ZQXRD
froid	FROID	GLACIAL	FRAIS	QSDFG
glacial	GLACIAL		FROID / FRAIS	NRFXTZS
acceptable	ACCEPTABLE	BON / EXCELLENT		KRPQFXSVN
bon	BON	EXCELLENT	ACCEPTABLE	ZKP
excellent	EXCELLENT		BON / ACCEPTABLE	PTRZQMBFX
