# Supplementary material for: Scalar Implicatures: The Psychological Reality of Scales
Source: Front Psychol. 2016 Oct 25;7:1500. doi: 10.3389/fpsyg.2016.01500 (PMC5078746; doi:10.3389/fpsyg.2016.01500)
Supplement: Supplementary file 2 [file Presentation_1.pdf]

<u>Supplementary materials</u>

The reviewers of this paper were concerned by the fact that we have tested a somewhat heterogeneous set of lexical scales, consequently they recommended to plot the results obtained for each lexical scale and verify whether they all behave as expected or whether there is variation in the expected results. However, given that for each subject there was only one data point per scale in each condition, a statistical analysis by scale would be meaningless. Thus, we refrain from providing a full interpretation of what differences between scales would mean given that these differences may not be reliable. Nevertheless, we looked at the results by scale and we present these data below, only for an exploratory purpose. Figure S1 represents the averages of the priming effect obtained for each scale across all participants (i.e., reaction time in the *Implication* condition minus reaction time in the *Implicature* condition averaged over all participants).

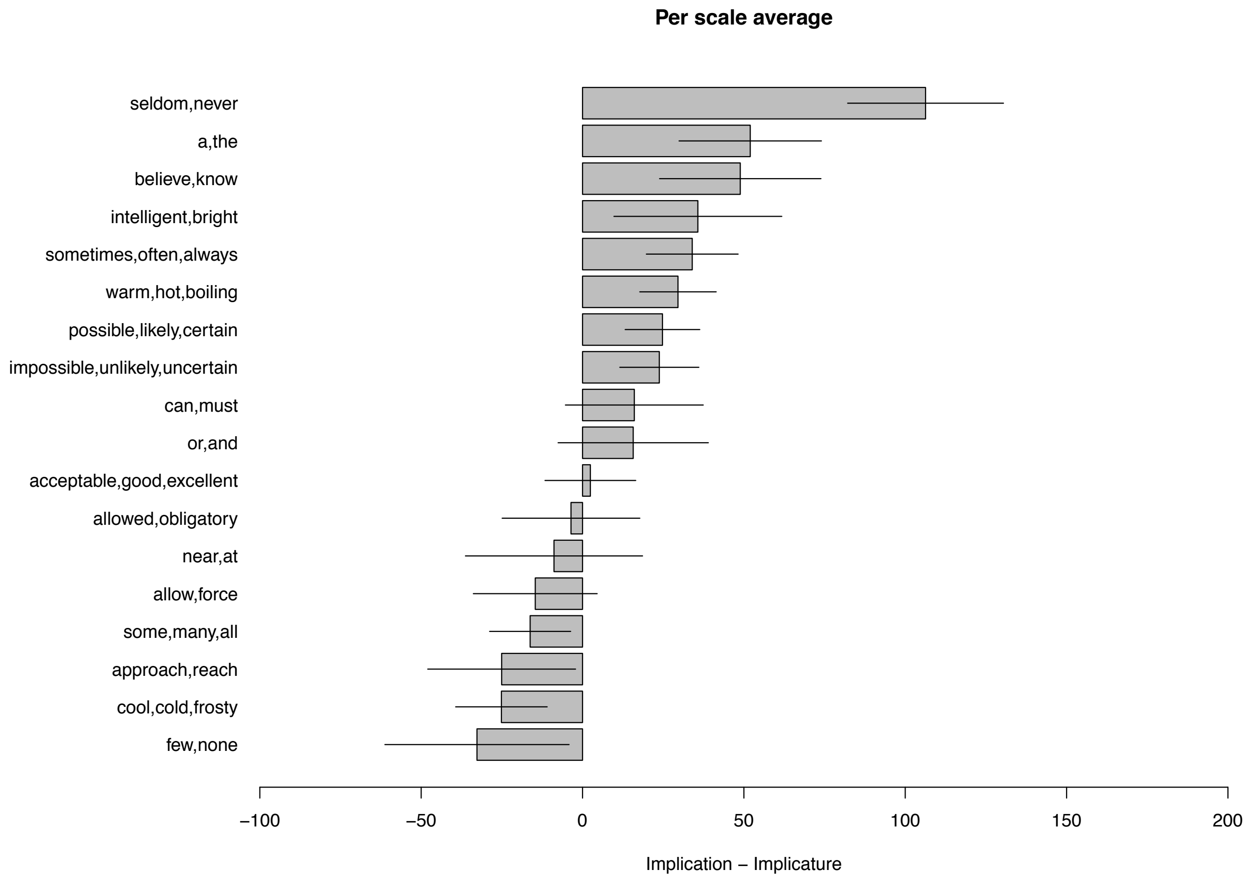

Figure S1: Per scale average of the contrast in reaction time between Implication and Implicature conditions in milliseconds. Error bars stand for the standard error of the mean.

The reviewers proposed that some scales tested might induce pragmatic interpretations at a much higher rate than others. For instance, they suggested that the effect generated by scales with logic connectives (<*and, or*>), quantifiers (<*some, all*>) and modals (<*allowed, obligatory*>) could be stronger than other scales such as <*bright, intelligent*>. Supporting this hypothesis, van Tiel et al. (2016) have argued, based on experimental investigations, that some scales (notably <*all, many, some*> and <*and, or*>) induce a much higher rate of pragmatic interpretations for scalar implicatures than do others (e.g., <*small, tiny*>). Thus, it would be important to explore our data pattern more closely and examine whether all the scales tested show the same effect size or not.

As depicted in Figure S1, the important priming effect we have found from

Implication minus Implicature condition can be observed for the majority of the lexical scales tested, although in some cases this effect was absent or tended to be in favor of the Implication condition.

In order to address the question about the heterogeneity of the scales tested and the possibility that we may observe different pattern of results for different kinds of lexical scales, in Figure S2 we also present the effect by scale following the classification of scales proposed by van Tiel et al., (2016). Van Tiel and colleagues, distinguishes between Bounded and Unbounded scales (those that do not fall clearly into this classification were termed "Neutral"). Scales are bounded when the stronger term in the scale cannot be replaced by an even stronger one (e.g., in the scale <*some, many, all*>, there is no term stronger than *all*). They are unbounded otherwise.

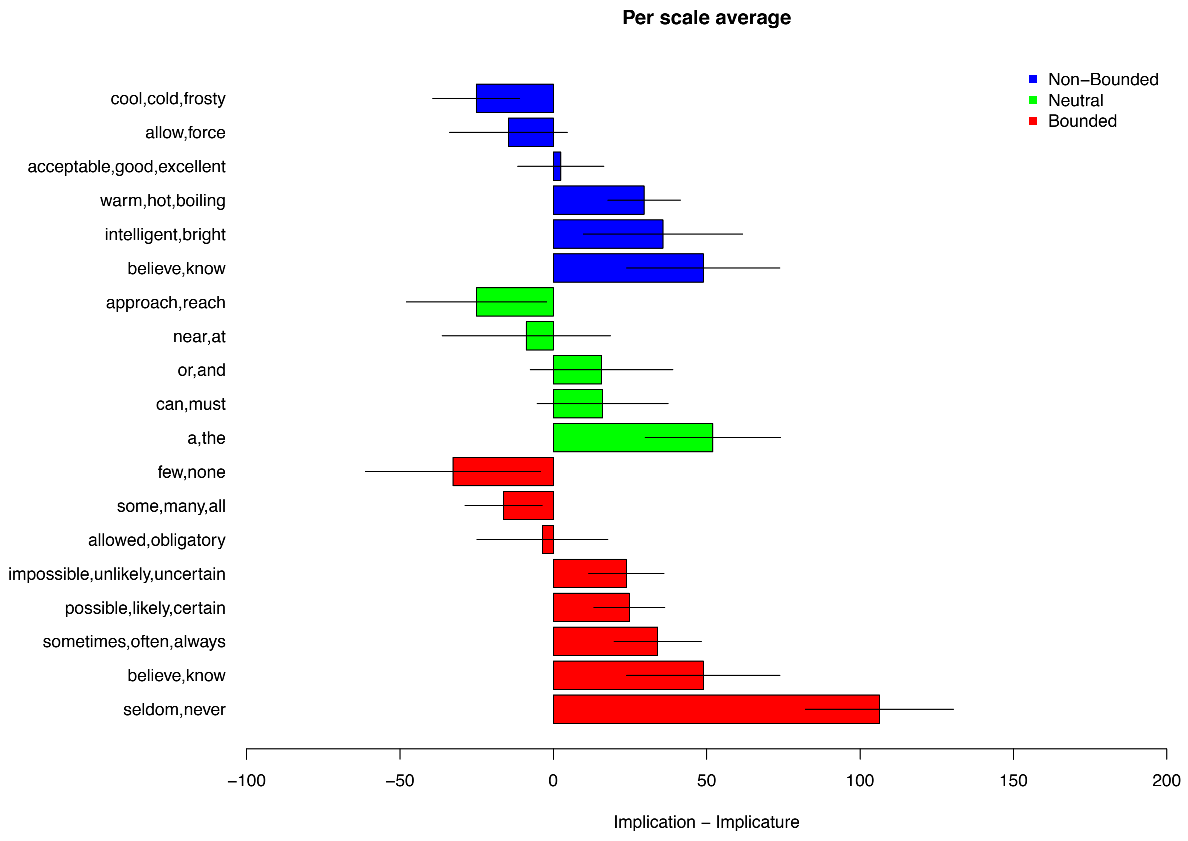

Figure S2: Per scale average of the contrast in reaction time between Implication and Implicature conditions in milliseconds split by type of lexical scalar implicature scales proposed by van Tiel et al. (2016). Blue bars represent Non-Bounded lexical scales, Green bars represent Neutral lexical scales and Red bars represent Bounded lexical scales. Error bars stand for the standard error of the mean.

As can be observed in Figure S2, no clear effect of Van Tiel's distinction between Bounded and Unbounded scales is observed. In other words, the same overall variability is seen in each classification of scales. So no clear pattern for scales type emerges. Note however, that since we investigated the priming effect of the Implicature condition vs. the Implication condition across all the scales, and we had no prior hypothesis regarding the magnitude of effects that would be triggered by each single scale or type of scales, our data does not allow us to propose an interpretation of the effects derived by each scale individually. However, it might be considered that since the main result of this paper (i.e., the asymmetry in reaction time between the implication and the implicatures conditions) can be observed even into a rather heterogeneous set of lexical scales, this seems to be a robust finding. This effect argues for a general mechanism, which may be either on the lines of Chierchia's neo-Gricean syntactic account, or on those of Geurts' pragmatic Gricean account. Nevertheless, our results are entirely compatible with the idea that scales may differ in how strongly they mandate pragmatic interpretations, or in the degree of automaticity with which they are accessed in the interpretation of scalar implicatures. Further studies can take our study as a starting point and implement our design in order to be able to explore the question of how and why some lexical scales can behave differently in they way they induce pragmatic interpretations.

References:

van Tiel, B., van Miltenburg, E., Zevakhina, N., Geurts, B. (2016). Scalar diversity. *Journal of Semantics*, 33(1), 107-135.
